# A controlled study of Gd-EOB-DTPA-enhanced MRI compared with enhanced CT in assessing lesion status after TACE for hepatocellular carcinoma

**DOI:** 10.3389/fmed.2025.1602428

**Published:** 2025-07-25

**Authors:** Qichao Cheng, Xuewen Peng, Zhanyu Zhou, Bowen Wang, Jinlei Fan, Liping Zuo, Weiwei Lv, Dexin Yu

**Affiliations:** Department of Radiology, Qilu Hospital of Shandong University, Jinan, Shandong, China

**Keywords:** hepatocellular carcinoma, transcatheter arterial chemoembolization, Gd-EOB-DTPA-enhanced MRI, contrast-enhanced CT, lesion status

## Abstract

**Objective:**

This study aims to evaluate the diagnostic capability of Gd-EOB-DTPA-enhanced MRI in assessing lesion status following transcatheter arterial chemoembolization (TACE) for hepatocellular carcinoma (HCC), in comparison to contrast-enhanced CT (CECT).

**Methods:**

A total of 56 patients with HCC who underwent Gd-EOB-DTPA-enhanced MRI and CECT scans post-TACE were initially enrolled. The ability of both imaging modalities to differentiate between surviving, new, or necrotic lesions was assessed, using digital subtraction angiography (DSA) or interventional diagnostic results as the reference standard. Detection rates were compared using the chi-square test, while sensitivity, specificity, and accuracy were analyzed with McNemar’s test.

**Results:**

After applying inclusion and exclusion criteria, 48 patients were eventually included in the analysis. The reference standard identified 14 cases of surviving lesions, 19 of new lesions, and 15 of necrotic lesions. Gd-EOB-DTPA-enhanced MRI demonstrated a sensitivity of 93.9% (31/33), specificity of 100% (15/15), and a Youden index of 0.939, whereas CECT exhibited a sensitivity of 51.5% (17/33), specificity of 60.0% (9/15), and a Youden index of 0.115.

**Conclusion:**

The findings indicate that Gd-EOB-DTPA-enhanced MRI possesses superior diagnostic value for evaluating lesion status in HCC post-TACE compared to CECT, as evidenced by significant differences in sensitivity and specificity (*p* < 0.05).

## 1 Introduction

Hepatocellular carcinoma (HCC) is the most common pathological type of primary liver cancer and occurs often associated with chronic liver disease and cirrhosis (1, 2). According to statistics, the new cases and deaths of liver cancer have reached 860,000 and 750,000 in 2022 (3), with HCC accounting for a high proportion of 75 ~ 85%. The incidence is expected to exceed 1 million people in 2025 (4). About 70% of patients are already in the middle and advanced stages when their lesions are first detected, which is tantamount to increasing the burden of disease diagnosis and treatment (5). Transcatheter arterial chemoembolization (TACE) is one of the loco-regional treatments for unresectable HCC. However, due to factors such as the rich blood supply of HCC, the postoperative recurrence rate is often high. So regular follow-up to clarify the status of lesion survival and recurrence is crucial (6, 7).

Currently, Computed Tomography (CT) and Magnetic Resonance Imaging (MRI) are the main clinical imaging tools after TACE. CT can evaluate the lesion according to the deposition state of lipiodol, and the accumulated lipiodol suggests tumor necrosis; however, CT is difficult to penetrate through the high-density lipiodol deposition area, making it unable to accurately diagnose the residual tumor tissues that have been covered up (8). MRI is not affected by lipiodol, and it can carry out stereoscopic imaging in multiple planes, and it has a higher sensitivity and accuracy in the detection of postoperative lesions; however, for the hypovascular tumors with insignificant enhancement, there is a high risk of missed diagnosis (9).

Contrast-enhanced CT (CECT) and Gd-EOB-DTPA-enhanced MRI has overcome the shortcomings of the above methods. Gadolinium Ethoxybenzyl Diethylenetriamine Pentaacetic Acid (Gd-EOB-DTPA), a hepatobiliary-specific MRI contrast agent, can enhance dynamic imaging of high-vessel HCC in the arterial phase, improve the diagnosis of low-vessel HCC in the hepatobiliary phase, providing a more reliable basis for postoperative efficacy evaluation (10, 11). However, it is unclear whether there is a difference between CECT and Gd-EOB-DTPA-enhanced MRI in the diagnosis of HCC lesions after TACE. In this study, we explored the effectiveness of Gd-EOB-DTPA-enhanced MRI in the assessment of lesion status after TACE using CECT as a control with particular focus on the detection and characterization of new lesions. A subgroup analysis was additionally performed to compare the assessment efficacy between Child-Pugh class A and B patients.

## 2 Materials and methods

### 2.1 Clinical data

This prospective study was endorsed by the Ethics Committee of Qilu Hospital (Ethics No. QLYY-KY-2021-231), and all patients signed an informed consent form. Our team adhered to the ethical guidelines of the Declaration of Helsinki and was firmly committed to patient privacy. The study included a total of 56 patients who underwent CECT and Gd-EOB-DTPA-enhanced MRI examinations after receiving TACE treatment at Qilu Hospital between January 2022 and December 2023. This study was based on an expected difference in diagnostic accuracy between MRI (90%) and CT (60%), combined with a 6-month recurrence rate of 50% after TACE. Under a significance level of *α* = 0.05 and a statistical power of 80%, a total of 56 patients were ultimately enrolled (12).

### 2.2 Inclusion and exclusion criteria

#### 2.2.1 Inclusion criteria

(1) HCC (based on CT or MRI diagnostic results) patients aged ≥ 18 years; (2) Patients receiving TACE for the first time and planning for long-term follow-up; (3) Liver function classified as Child-Pugh grade A or B, with an ECOG score of 0–2 points; (4) The patient can undergo Gd-EOB-DTPA enhanced MRI and CECT scan.

#### 2.2.2 Exclusion criteria

(1) Pregnant or lactating female patients; (2) Patients with Child-Pugh grading of C and severe liver function impairment; (4) Patients with class hypersensitivity or allergic reactions to contrast agents or with significant hyperthyroidism; (5) Patients unable to undergo MRI examination due to claustrophobia or presence of implants that cannot be approached by strong magnetic fields; (6) Failure to complete key examination procedures (e.g., DSA).

### 2.3 Imaging examination methods

#### 2.3.1 Gd-EOB-DTPA enhanced MRI scanning method

The MRI scanner (Discovery 750w, GE Healthcare, United States) used a 3.0 T MR instrument with a 48-channel phased array coil in the abdomen to examine the patient. Before administering Gd-EOB-DTPA (Bayer-Schering, Germany, Promethean), the following imaging sequences were acquired: T2-weighted images (T2WI) during breath-hold, in-and-out phase T1-weighted image (T1WI, LAVA-Flex sequence), and masked processing image (LAVA sequence) (13, 14). After the intravenous administration of saline and 0.1 mL/kg of Gd-EOB-DTPA, the following sequences should be collected in sequence: arterial phase (20–25 s), portal venous phase (1 min) and transitional phase (3–5 min) transverse-axial T1WI (LAVA sequence), axial fat-suppressed T2WI sequences, resonance Diffusion-Weighted Imaging (DWI) and hepatobiliary phase (20 min) coronal and axial T1WI images. The total time taken by the patient to perform a scan was approximately 25 min and the parameters of the scanning sequences involved in this procedure are shown in Table 1.

**Table 1:** Gd-EOB-DTPA-enhanced MRI scanning parameters.

Scanning sequences	FOV (mm^2^)	Slice thickness (mm)	Slices	Scanning matrix	NEX	Bandwidth (Hz)
T2WI	360 × 320	6	28	320 × 224	2	62.50
LAVA-Flex	380 × 304	5	46	288 × 172	1	90.91
LAVA	380 × 304	5	46	320 × 224	1	83.33
T2WI fs	360 × 320	6	28	320 × 224	2	62.50
DWI	360 × 320	6	28	128 × 130	2	250.0

T2WI, T2-weighted images; T2WI fs, fat-suppressed T2-weighted images; DWI, Diffusion-Weighted Imaging.

#### 2.3.2 CECT scanning method

A routine upper abdominal CT scan (Discovery CT750, GE) was performed first, followed by scanning at the arterial phase (35 s), venous phase (60 s), and delayed phase (2–3 min) after drug injection (300 mg I/mL non-ionic iodine contrast agent Ultravist, Germany Bayer-Schering). It was recommended to prioritize CT examination first, with an interval of at least 24 h before performing the MRI scan.

### 2.4 Assessment of post-TACE efficacy

#### 2.4.1 Reference standard

This longitudinal investigation was conducted within the context of TACE necessitating serial follow-up assessments. Given the ethical constraints associated with obtaining pathological confirmation at every follow-up for all participants, we established a hierarchical diagnostic protocol: Lesion evaluation was primarily conducted by interventional radiologists through Digital Subtraction Angiography (DSA) imaging, whereas cases demonstrating equivocal DSA findings underwent supplementary pathological verification by clinical specialists. These combined diagnostic outcomes constitute the diagnostic reference standard. Qualitative analysis yielded three possible outcomes: (1) Necrotic lesions after TACE (no activity): No tumor enhancement was observed on DSA, or pathological biopsy showed no viable HCC cells; (2) Surviving lesion after TACE: Persistent tumor enhancement was detected on DSA, or pathological biopsy confirmed HCC; (3) New lesion after TACE: A new lesion after TACE was defined as a newly detected enhancing nodule in an untreated area on DSA, or a newly developed HCC lesion confirmed by histology. Based on these results, clinical treatment plans were made, and follow-up continued until new, surviving, or residual lesions were identified, at which point the study was terminated (residual lesions include both surviving and new lesions).

#### 2.4.2 Radiological assessment

Two radiologists independently performed a blinded review of the films to record the validity indexes of hepatocellular carcinoma lesions in the images before and after Gd-EOB-DTPA enhanced MRI and CECT scans. Our analytical strategy employed Liver Imaging Reporting and Data System (LI-RADS) (15) to characterize individual lesion activity through Gd-EOB-DTPA-MRI and CECT findings, with particular emphasis on detecting surviving HCC in postoperative settings. However, recognizing the intrinsic limitation of LI-RADS in evaluating multifocal disease progression, we have synergistically integrated Modified Response Evaluation Criteria in Solid Tumors (mRECIST) (16) methodology. This complementary approach enabled systematic surveillance of global disease status, including sensitive detection of new lesions.

### 2.5 Statistical analysis

The expression form of count data was mean ± standard deviation, and percentage (%) could be used for measurement data. The number of cases consistent with the reference standard diagnostic results between Gd-EOB-DTPA enhanced MRI and CECT scans was analyzed, and thus the accuracy rate was calculated: accuracy rate = number of Gd-EOB-DTPA-enhanced MRI or CECT diagnoses/number of DSA or interventional diagnoses, and the chi-square test was applied for comparison. Kappa tests were used to assess the consistency of opinions between the two reviewers, with Kappa ≥ 0.7 indicating good concordance; in cases of poor agreement, a third senior radiologist performed a blinded review and arbitration. To assess the consistency of Gd-EOB-DTPA-enhanced MRI and CT in evaluating surviving, new, and necrotic lesions against the reference standard, we employed a combined approach using LI-RADS and mRECIST. Detection rates were calculated, and the chi-square test was applied to compare the detection capabilities of the two imaging methods for surviving and new lesions. Sensitivity and specificity were derived from true-positive (TP), false-positive (FP), true-negative (TN), and false-negative (FN) rates, along with corresponding Youden indices (Youden index = sensitivity + specificity − 1). Data were analyzed using SPSS 26.0. McNemar’s test comparison was used to compare sensitivity and specificity. All differences were considered statistically significant at *p* < 0.05 in this study.

## 3 Analysis of results

### 3.1 Analysis of general clinical data of patients

**Figure 1:**
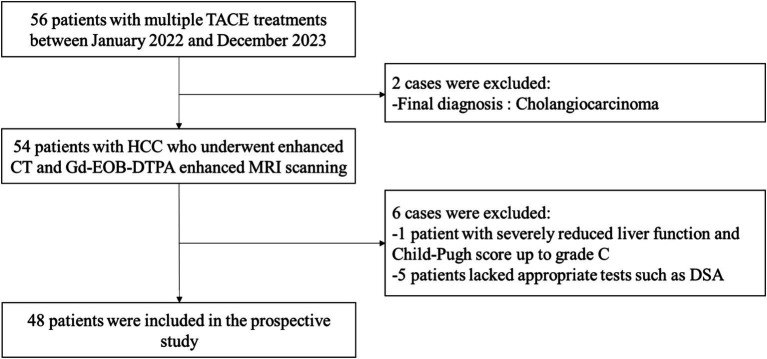
The flow chart of the study.

The clinical diagnosis of the selected 56 patients with HCC showed that a total of 8 cases did not meet the study criteria and were excluded: 1 patient had decreased liver function and Child-Pugh score up to grade C; 2 patients were finally diagnosed with cholangiocarcinoma; and 5 patients lacked the appropriate examinations such as DSA scan. Therefore, a total of 48 patients with HCC who had undergone TACE treatment were finally included. The flow chart of the study is shown in Figure 1. The basic clinical information is shown in Table 2. Kappa test results indicated strong consistency between the two reviewers (Kappa = 0.83).

**Table 2:** Clinical baseline data of included patients.

Clinical information (*n* = 48)		Statistical value
Age		59.2 ± 8.0 (41~78)
Sex	Male	45 (93.8%)
	Female	3 (6.2%)
Cirrhosis	Yes	33 (68.8%)
	No	15 (31.3%)
Hepatitis virus infection	Hepatitis B infection	41 (85.4%)
	Hepatitis C infection	2 (4.2%)
	Other infections	5 (10.4%)
Alcoholism	Yes	18 (37.5%)
	No	30 (62.5%)
Non-alcoholic fatty liver	Yes	27 (56.3%)
	No	21 (43.7%)
Child-Pugh	A	38 (79.2%)
	B	10 (20.8%)

### 3.2 LI-RADS and mRECIST evaluation results

We applied LI-RADS criteria to evaluate HCC treatment response, with comparative analysis of imaging modalities conducted across three diagnostic classification tiers. The findings revealed suboptimal detection rates for new lesions, as quantified by Gd-EOB-DTPA-enhanced MRI (73.7%) and CECT (52.6%). This phenomenon may stem from LI-RADS emphasis on individual lesion characterization rather than holistic tumor burden evaluation, particularly in identifying new lesions. To mitigate this limitation, we implemented mRECIST criteria for post-TACE assessment (Supplementary Table 1), with focused stratification of PD cases and new lesions. By integrating both LI-RADS and mRECIST, we assessed whether HCC remained active post-treatment or if new lesions had developed, thereby enabling comprehensive characterization of treatment responses.

### 3.3 Detection ability of Gd-EOB-DTPA-enhanced MRI and CECT

According to the reference standard, post-TACE evaluation identified 14 cases of surviving lesions, 19 new lesions, and 15 necrotic lesions. Gd-EOB-DTPA-enhanced MRI detected 13 out of 14 surviving lesions, 18 out of 19 new lesions, and all 15 necrotic lesions. In contrast, CECT identified 8 surviving lesions, 9 new lesions, and 9 necrotic lesions. The detection rates for new tumors were 94.7% for MRI and 47.4% for CT, showing a significant difference in their ability to detect new lesions (*p* < 0.01), with MRI demonstrating superior performance. The detailed results are presented in Table 3 (Supplementary Figure 1).

**Table 3:** Analysis of the detection ability of Gd-EOB-DTPA-enhanced MRI and CECT.

Lesion status	Reference standard	Gd-EOB-DTPA-enhanced MRI		CECT	
		Diagnoses	Detection rate	Diagnoses	Detection rate
Surviving lesions	14	13	92.9%	8	57.1%
New lesions	19	18	94.7%	9	47.4%
Necrotic lesions	15	15	100%	9	60.0%

Gd-EOB-DTPA, Gadolinium ethoxybenzyl diethylenetriamine pentaacetic acid; CECT, contrast-enhanced CT.

### 3.4 Efficacy evaluation of Gd-EOB-DTPA-enhanced MRI and CECT post-TACE

As shown in Supplementary Table 2, Gd-EOB-DTPA-enhanced MRI scans aligned with DSA or interventional diagnosis results in 46 cases, with only 2 missed cases, while CECT detected 26 cases confirmed by the reference standard, missing 22 cases. The accuracy rates for Gd-EOB-DTPA-enhanced MRI and CECT were 95.8 and 54.2%, respectively. Chi-square tests (*p* < 0.01) revealed a significant difference in diagnostic ability between the two methods following TACE, with Gd-EOB-DTPA-enhanced MRI yielding higher accuracy.

### 3.5 Sensitivity and specificity analysis

The sensitivity of Gd-EOB-DTPA-enhanced MRI for the diagnosis of residual tumor lesions was further calculated to be 93.9% (31/33) and the specificity was 100% (15/15) in the study, the Youden index 0.939. The sensitivity and specificity of CECT for the diagnosis of residual tumor lesions were 51.5% (17/33) and 60.0% (9/15), the Youden index 0.115, respectively. McNemar’s test indicated significant differences between the two modalities (*p* < 0.05), affirming that Gd-EOB-DTPA-enhanced MRI outperformed CECT in evaluating postoperative HCC tissue status. The final statistical results can be seen in Table 4.

**Table 4:** Diagnostic results of residual lesions by Gd-EOB-DTPA-enhanced MRI and CECT.

Examination method	True positive tp	False positive tn	True negative fp	False negative fn	Sensitivity	Specificity
Gd-EOB-DTPA-enhanced MRI	31	0	15	2	93.9%	100%
CECT	17	6	9	16	51.5%	60.0%

Gd-EOB-DTPA, Gadolinium ethoxybenzyl diethylenetriamine pentaacetic acid; CECT, contrast-enhanced CT.

### 3.6 Consistency analysis under Child-Pugh grade

Table 5 presents the concordance between Gd-EOB-DTPA-enhanced MRI and CECT with the reference standard under the Child-Pugh classification. A significant difference was observed between the two imaging methods in patients with grade A (*p* < 0.01), while no such difference was found in grade B. However, the small sample size in grade B may have introduced some degree of error.

**Table 5:** Consistency with reference standard under Child-Pugh.

Child-Pugh	Gd-EOB-DTPA-enhanced MRI	CECT	*p* value
A	97.4% (37/38)	52.6% (20/38)	**p < 0.01**
B	90.0% (9/10)	60.0% (6/10)	0.30

Gd-EOB-DTPA, Gadolinium ethoxybenzyl diethylenetriamine pentaacetic acid; CECT, contrast-enhanced CT. The level of significance was set as *p* < 0.05.

### 3.7 Imaging scan analysis

**Figure 2:**
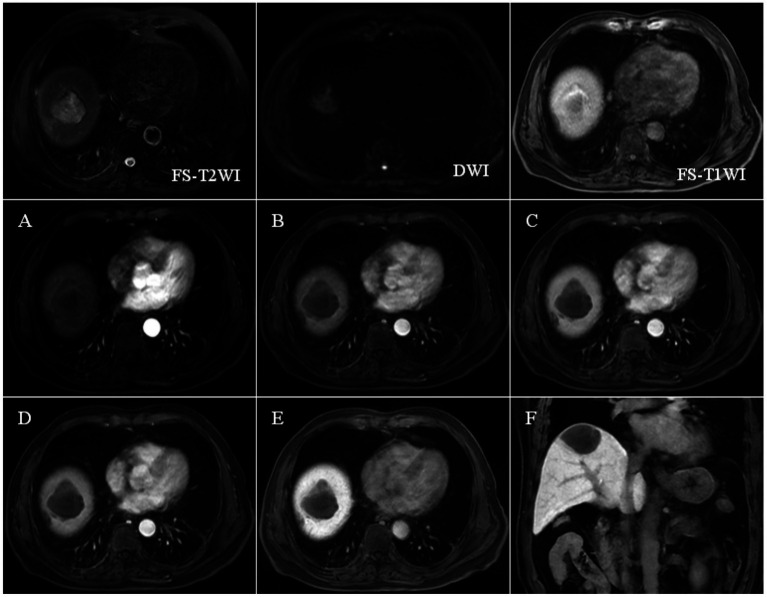
FS-T2WI and DWI: patchy high signal at the top of the right lobe of the liver: FS-T1WI: patchy high signal is seen at the top of the right lobe of the liver, and a patchy slightly low signal is seen within it. Panels **(A–F)** show the early arterial, late arterial, portal venous, delayed, hepatobiliary axial and coronal positions. EOB-MRI enhancement scans can accurately show the enhancement of the lesion after TACE: the necrotic area shows high signal in T1WI, low signal in T2WI and DWI, and the enhancement scans do not show any enhancement, and the hepatobiliary phase shows a low signal, which is a manifestation of coagulative necrosis.

**Figure 3:**
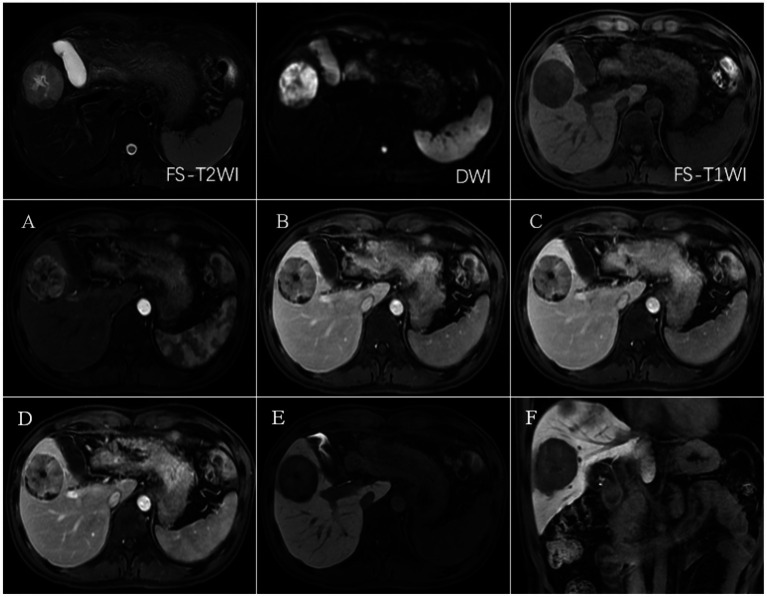
FS-T2WI and DWI: rounded high signal in the right lobe of the liver, with a diameter of about 48.9 mm; FS-T1WI: rounded low signal in the right lobe of the liver. **(A–F)** show the early arterial, late arterial, portal venous, delayed, hepatobiliary axial and coronal positions. EOB-MRI enhancement scan can accurately show the enhancement of the lesion after TACE: the surviving area shows increased signals in T2WI and DWI, and the enhancement scan shows the characteristics of “fast-in-fast-out” enhancement, with low signals in the hepatobiliary phase. The surviving areas showed increased T2WI and DWI signals, with “fast-in-fast-out” enhancement characteristics and low signals in the hepatobiliary phase. The necrotic area showed high signal in T1WI, low signal in T2WI and DWI, no enhancement in the enhanced scan, low signal in the hepatobiliary phase, and coagulative necrosis.

**Figure 4:**
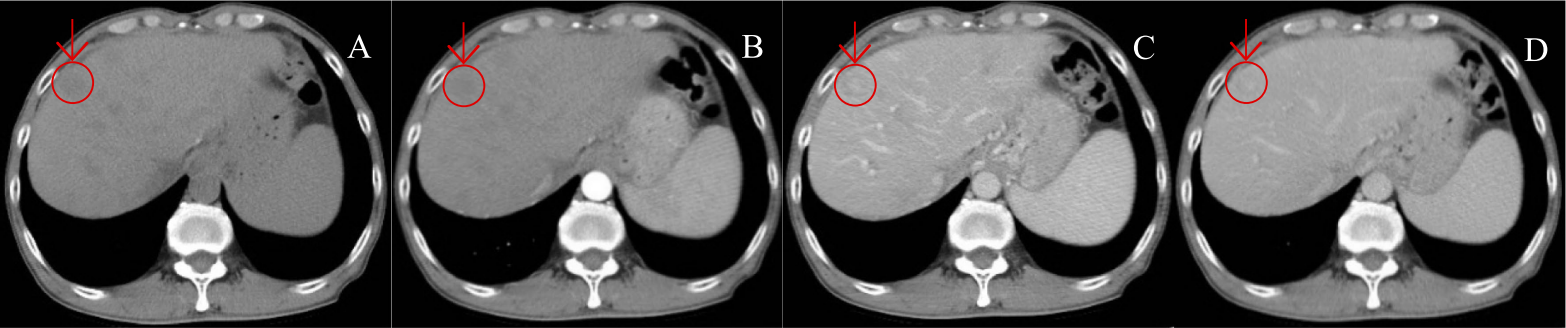
A patient with HCC was reviewed after TACE treatment. **(A–D)** show the images of CT plain **(A)** and enhanced scans of arterial phase **(B)**, portal vein **(C)**, and delayed phase **(D)**, in sequence. CT plain scan: New lesions were seen in the hepatic parenchyma, with nodular hypodensity, the large one was about 19.6 mm in diameter; enhancement scan showed insignificant enhancement in arterial phase, slightly hypodense in portal vein and delayed phase, and hyperdense in the center, which was atypical of “fast-in-fast-out” performance.

**Figure 5:**
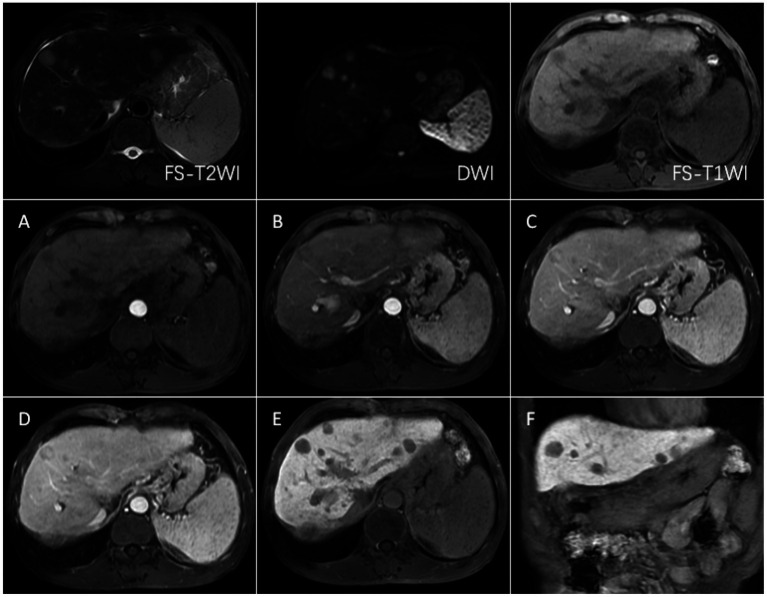
FS-T2WI and DWI: several round-like T2WI and DWI high-signal nodules were seen in the left and right lobes of the liver, with the largest being about 19.6 mm in diameter; FS-T1WI: several round-like low-signal nodules were seen in the left and right lobes of the liver. **(A–F)** show the early arterial, late arterial, portal venous, delayed, hepatobiliary axial and coronal positions. MRI dynamic scan: the lesion did not have definite enhancement in the early arterial stage, and it showed inhomogeneous and mild enhancement in the late arterial stage, with low-signal edges in the portal vein stage and the delayed stage, and high-signal in the central area; Hepatobiliary phase—axial and coronal: the lesion showed low signal, no contrast uptake was seen, suggesting that the multiple intrahepatic nodules were multiple HCC.

CECT scans were compromised by high-density lipiodol deposits, obscuring imaging clarity. In contrast, Gd-EOB-DTPA-enhanced MRI showed high signals during the arterial phase and low signals in the hepatobiliary phase for surviving and new lesions. The results of complete necrosis scans are shown in Figure 2 (Supplementary Figure 2); the results of partial survival scans are shown in Figure 3 (Supplementary Figure 3); and the results of new lesion scans are shown in Figures 4, 5.

## 4 Discussion

Our research results indicate that Gd-EOB-DTPA-enhanced MRI has significantly superior diagnostic value compared to CECT for assessing post-TACE lesion status. Specifically, the detection rate of new HCC lesions with Gd-EOB-DTPA-enhanced MRI (94.7%) is much higher than that of CECT (47.4%), and it provides higher sensitivity, specificity, and accuracy for clinical diagnosis. These results highlight the clear advantage of Gd-EOB-DTPA-enhanced MRI, particularly in the detection of new lesions (14), reinforcing its value in clinical practice. Studies suggest LI-TR-based EOB-MRI demonstrates comparable efficacy to CECT in tumor response assessment (17). This contrasts with our findings, likely because LI-TR prioritizes macroscopic viable tumors over emergent microscopic lesions.

The study shows that the detection rates of Gd-EOB-TPA-enhanced MRI on surviving, new and necrotic lesions are 92.9% (13/14), 94.7% (18/19) and 100% (15/15) respectively, with high sensitivity and specificity. This indicates that this imaging method has a good ability to evaluate the efficacy of the treatment after TACE. Gd-EOB-DTPA enhanced MRI can be used to clearly reflect the differences in hemodynamics between the tumor and normal tissue, and perform dynamic imaging of highly vascular hepatocellular carcinoma during the arterial phase (11). The injected Gd-EOB-DTPA can be taken up by normal hepatocytes through the organic anion transporting polypeptide 1B3 (OATP1B3), which can show higher signals on the images (10); whereas, the surviving and new tumor tissues lack the expression of the related transport peptide 1B3, have poorer ability to uptake Gd-EOB-DTPA, then show lower signals in the hepatobiliary phase, forming a contrast with the surrounding normal tissue (10, 13, 18, 19). Therefore, based on the significant signal differences between tissues in the image, Gd-EOB-DTPA enhanced MRI greatly improves the detection rate of low vascular HCC.

CECT, by intravenously injecting contrast agent, is used to develop and analyze the status of tissue blood supply. Tumor tissue, which has abundant arterial blood supply, shows significant enhancement and forms a sharp contrast with the surrounding normal tissue (20), thus it can be used as an imaging standard to determine the viability of HCC after TACE. However, the study found that the detection rates of CECT for surviving, new, and necrotic lesions are 57.1% (8/14), 47.4% (9/19), and 60.0% (9/15) respectively. The final results are all lower than those of Gd-EOB-DTPA enhanced MRI. This may be due to the fact that CECT has a poorer diagnostic effect for low vascular small hepatocellular carcinomas: the blood supply is insufficient and the imaging is inconspicuous, which appear to be missed (21, 22). Also, CT scans are susceptible to deposition of lipiodol.

Among Child-Pugh A and B subgroups, Gd-EOB-DTPA-enhanced MRI demonstrates significantly higher concordance with the reference standard compared to CECT in subgroup A (*p* < 0.01). However, no statistical significance is observed in subgroup B, potentially due to diminished contrast differentiation of HCC lesions caused by reduced hepatic background signal in the hepatobiliary phase following impaired liver function (23), compounded by the limited sample size in subgroup B. Another study reports extracellular contrast agents outperform EOB-MRI (14), potentially attributable to their focus on populations with impaired hepatic function, whereas most subjects in our cohort are Child-Pugh class A. Furthermore, our study demonstrates that EOB-MRI exhibits distinct advantages in detecting and characterizing new lesions.

It is noteworthy that the study results show that Gd-EOB-DTPA enhanced MRI also has cases of missed diagnoses., which may be related to the fact that a small proportion of HCCs still have the ability to uptake Gd-EOB-DTPA, according to the relevant literature (24). The expression level of the OATP1B3 transporter is closely related to the degree of cell differentiation: the higher the degree of differentiation, the higher the expression level and the stronger the uptake capacity (24–26). Therefore, well-differentiated HCC may exhibit signal intensity similar to that of the surrounding normal liver tissue on imaging (19). Therefore, well-differentiated lesions are difficult to distinguish in the images, leading to underdiagnosis and misdiagnosis, which reduce the detection rate of Gd-EOB-DTPA enhanced MRI for residual and new cancerous lesions.

In certain clinical scenarios, Gd-EOB-DTPA-enhanced MRI may not be prioritized as the first-line diagnostic approach. This includes situations where the higher initial cost of this contrast-enhanced imaging modality compared to conventional methods (27) creates financial barriers for patients, as well as cases where there is a history of significant adverse reactions to Gd-EOB-DTPA (28). CECT, characterized by its lower cost and widespread application, is more readily accepted by patients with clear diagnoses and uncomplicated conditions (27).

The present study has the following limitations. First, the sample size of cases included in this prospective study was small, which may result in selective bias; this study terminated enrollment upon achieving target sample size, and complimentary imaging protocols inadvertently led to exclusion of patients lacking essential examinations. To reduce the risk of bias, future studies may increase the sample size and adopt a stratified sampling method to ensure a balanced distribution of patients across different Child-Pugh classifications. Second, interventional diagnosis is not suitable for all HCC patients, and this study only involved cases with unclear DSA imaging. But DSA still has some shortcomings in evaluating the efficacy of post-TACE, and the results cannot completely replace interventional diagnosis. Third, there is a lack of specific analysis of the imaging results of hepatocellular carcinomas at different differentiation stages, which can affect the results of imaging scans. And in-depth study can help us better understand the reasons for the existence of the defects. Fourth, this study initially planned four follow-up rounds, but voluntary participation and the free service model resulted in low patient compliance. Participants often missed scheduled appointments, leading to inconsistent examination timelines and incomplete data collection across all planned phases, which significantly limited comprehensive analysis.

In summary, compared to CECT imaging, Gd-EOB-DTPA enhanced MRI can provide higher diagnostic value. On the one hand, Gd-EOB-DTPA enhanced MRI has better evaluation value for the efficacy of TACE, and its ability to distinguish residual and necrotic lesions is superior to CECT, reducing the risk of missed and misdiagnosed cases. On the other hand, Gd-EOB-DTPA-enhanced MRI is able to better detect hypovascular HCC. Previous studies have found that early HCCs usually present as low vascular lesions (29). From a healthcare economics perspective, investing in early diagnosis can reduce costs from extended hospital stays, complex treatments, and late-stage palliative care, ultimately leading to overall savings.

## Data Availability

The original contributions presented in the study are included in the article/Supplementary material, further inquiries can be directed to the corresponding authors.
