# Supplementary material for: A controlled study of Gd-EOB-DTPA-enhanced MRI compared with enhanced CT in assessing lesion status after TACE for hepatocellular carcinoma
Source: Front Med (Lausanne). 2025 Jul 25;12:1602428. doi: 10.3389/fmed.2025.1602428 (PMC12331694; doi:10.3389/fmed.2025.1602428)
Supplement: Supplementary file 1 [file Data_Sheet_1.docx]

**A controlled study of Gd-EOB-DTPA-enhanced MRI compared with enhanced CT in assessing lesion status after TACE for hepatocellular carcinoma**

**Page 8 Supplementary Fig.1** The results of Gd-EOB-DTPA-enhanced MRI and CECT

**Page 9 Supplementary Fig.2** A patient with HCC was reviewed after TACE treatment. Figures a-d show the images of CT plain (a) and enhanced scans of arterial phase (b), portal vein (c), and delayed phase (d), in sequence. Patchy high-density lipiodol deposits are seen in the tumor at the top of the right lobe of the liver on CT scanning.

**Page 9 Supplementary Fig.3** A patient with HCC was reviewed after TACE treatment. CT scan: patchy high-density lipiodol deposits were seen in the tumor of the right lobe of the liver. The arterial, portal venous and delayed stage lesions were still hyperdense.

**Page 8 Supplementary Table 1** mRECIST Evaluation Results

**Page 8** **Supplementary Table 2** Consistency analysis of scanning results with reference standard results


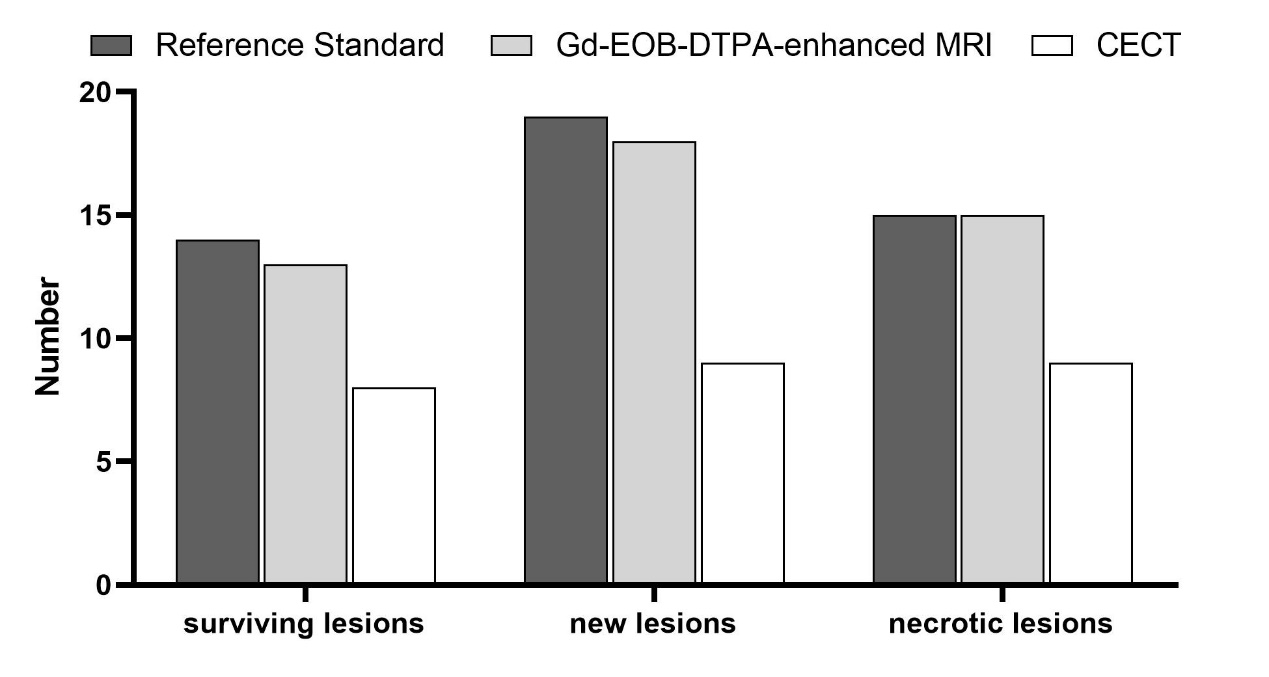


**Supplementary Fig.1** The results of Gd-EOB-DTPA-enhanced MRI and CECT


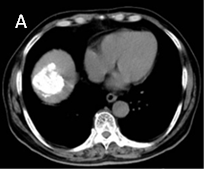

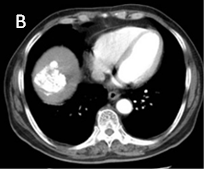

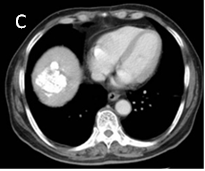

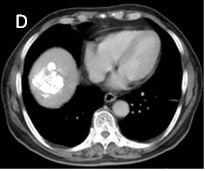


**Supplementary Fig.2** A patient with HCC was reviewed after TACE treatment. Figures a-d show the images of CT plain (a) and enhanced scans of arterial phase (b), portal vein (c), and delayed phase (d), in sequence. Patchy high-density lipiodol deposits are seen in the tumor at the top of the right lobe of the liver on CT scanning.


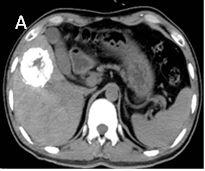

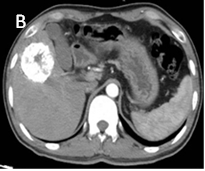

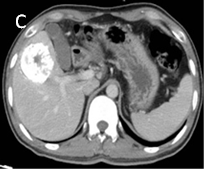

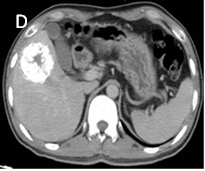


**Supplementary Fig.3** A patient with HCC was reviewed after TACE treatment. CT scan: patchy high-density lipiodol deposits were seen in the tumor of the right lobe of the liver. The arterial, portal venous and delayed stage lesions were still hyperdense.

**Supplementary Table 1** mRECIST Evaluation Results

| Examination Method | CR | PR | SD | **PD** |
| --- | --- | --- | --- | --- |
| Gd-EOB-DTPA-enhanced MRI | 16 | 10 | 3 | **19** |
| CECT | 10 | 6 | 23 | **9** |

**Supplementary Table 2** Consistency analysis of scanning results with reference standard results

| Examination Method | Diagnosis | Missed Diagnosis | Accuracy |
| --- | --- | --- | --- |
| Gd-EOB-DTPA-enhanced MRI | 46 | 2 | 95.8% |
| CECT | 26 | 22 | 54.2% |

*Note* CECT= Contrast-enhanced CT
